# Quantitative Risk Assessment for African Horse Sickness in Live Horses Exported from South Africa

**DOI:** 10.1371/journal.pone.0151757

**Published:** 2016-03-17

**Authors:** Evan S. Sergeant, John D. Grewar, Camilla T. Weyer, Alan J. Guthrie

**Affiliations:** 1 AusVet Animal Health Services, Canberra, Australian Capital Territory, Australia; 2 Veterinary Services, Western Cape Department of Agriculture, Elsenburg, South Africa; 3 Equine Research Centre, Faculty of Veterinary Science, University of Pretoria, Pretoria, South Africa; The University of Melbourne, AUSTRALIA

## Abstract

African horse sickness (AHS) is a severe, often fatal, arbovirus infection of horses, transmitted by *Culicoides* spp. midges. AHS occurs in most of sub-Saharan Africa and is a significant impediment to export of live horses from infected countries, such as South Africa. A stochastic risk model was developed to estimate the probability of exporting an undetected AHS-infected horse through a vector protected pre-export quarantine facility, in accordance with OIE recommendations for trade from an infected country. The model also allows for additional risk management measures, including multiple PCR tests prior to and during pre-export quarantine and optionally during post-arrival quarantine, as well as for comparison of risk associated with exports from a demonstrated low-risk area for AHS and an area where AHS is endemic. If 1 million horses were exported from the low-risk area with no post-arrival quarantine we estimate the median number of infected horses to be 5.4 (95% prediction interval 0.5 to 41). This equates to an annual probability of 0.0016 (95% PI: 0.00015 to 0.012) assuming 300 horses exported per year. An additional PCR test while in vector-protected post-arrival quarantine reduced these probabilities by approximately 12-fold. Probabilities for horses exported from an area where AHS is endemic were approximately 15 to 17 times higher than for horses exported from the low-risk area under comparable scenarios. The probability of undetected AHS infection in horses exported from an infected country can be minimised by appropriate risk management measures. The final choice of risk management measures depends on the level of risk acceptable to the importing country.

## Introduction

African horse sickness (AHS) is a severe arbovirus infection of horses and other equids, causing up to 95% case fatality rates in susceptible horses. African horse sickness virus (AHSV) is a member of the Orbivirus genus and family Reoviridae. Transmission is by *Culicoides* spp. biological vectors, primarily *C*. *imicola* and *C*. *bolitinos*, although other *Culicoides* spp. (Diptera, Ceratopogonidae) may also be capable vectors. There are nine serotypes of AHS virus and immunity is largely serotype specific. AHS is endemic in sub-Saharan Africa, with all nine serotypes occurring in many countries. Periodic outbreaks have occurred elsewhere, including northern Africa, the Middle East, the Asian sub-continent, Spain and Portugal [1–3].

The incubation period for AHS infection is 7–14 days, but may be as short as two days in severe infections [4]. Viraemia in challenged, vaccinated horses is detectable by PCR within 7 days [5]. The World Organisation for Animal Health (OIE) specifies the infective period (“the longest period during which an affected animal can be a source of infection”) in non-fatal AHS cases as 40 days [6].

Vaccination with attenuated live virus vaccines is the principal method of AHS control in endemic countries, while stamping out in combination with horse movement controls, vector control and use of inactivated vaccines are methods that have been used in non-endemic countries to control AHS [1]. As vaccines are based on live attenuated strains of AHSV, they are capable of establishing a transient infection in vaccinated animals, although whether or not vaccine infections are transmissible (as has been demonstrated for the closely related bluetongue virus) has not been confirmed, as is also the case with the possibility of reversion to virulence or genetic reassortment of vaccine strains [2–4, 7].

All nine serotypes of AHSV are present in South Africa, although specific serotypes in widespread circulation in the horse population vary from year to year. Although AHS is endemic in South Africa, outbreaks have a strong seasonality with most occurring from late spring through to autumn, associated with the period of greatest vector activity and abundance. Cases are also distributed geographically and temporally such that outbreaks start first in the north-east of the country each year and progress to the south and west as the *Culicoides* vector season progresses. The area around Cape Town and the Cape of Good Hope has historically had few outbreaks, usually in late summer or autumn and mostly thought to be associated with introduction of infected horses into the area at a time of high vector activity [3, 8].

AHS is a “listed” disease by the OIE, indicating its importance for animal health and international trade [9]. The OIE Code also provides recommendations in relation to AHS for countries wishing to safely import horses or other equids. These recommendations include provision for safe importation of equids from infected zones or countries through a combination of pre-export quarantine in a vector-protected quarantine facility and either serological or agent-identification testing while in quarantine [6]. However, very few countries have been prepared to accept imports from AHS-infected areas of South Africa using this protocol.

In 1997, an AHS free zone was described within the metropolitan area of Cape Town. The AHS controlled area, as defined in South African legislation, comprises the free zone, a surrounding surveillance zone of minimum 50 km width, where equine census and regular surveillance is undertaken, and a protection zone of minimum 100 km width, where AHS vaccination is compulsory (see S1 Fig for detailed maps). The free and surveillance zones are also protected against natural spread of virus from the rest of the country by geographic barriers provided by a mountain range and desert. Strict movement controls are applied for movements of equids into the controlled area to prevent incursions of AHS. Clinical surveillance has been successfully applied for early detection of incursions in both the surveillance and protection zones [8, 10].

The AHS free zone was originally established to facilitate export of live horses through a vector-protected quarantine facility at Kenilworth Racecourse in Cape Town. However, AHS outbreaks in the AHS controlled area (not in the free zone) in 1999, 2004, 2006, 2011, 2013 and 2014 have significantly disrupted trade, so that exports were only possible about 50% of the time between 1997 and 2014. The outbreak in 2006 occurred in the protection zone, while all other outbreaks were either within or intersected the surveillance zone. No outbreaks have ever occurred in the free zone. Although outbreaks have occurred in the surveillance and protection zones, they have been localised and rapidly contained and eliminated [8, 10]. These outbreaks were generally suspected of being due to illegal introduction of horses into the surveillance and protection zones [8].

During the five years from 2007 to 2011, while the AHS free zone was internationally recognised, a total of 480 horses were exported to Europe and other destinations through Kenilworth Quarantine Station. Annual throughput ranged from 27 to 189 per year and averaged 142 horses per year, with numbers reduced in 2007 and 2011 in particular due to AHS outbreaks. Since the 2011 outbreak in the surveillance zone exports have only been possible to Mauritius, with 657 horses exported between 2012 and 2014, averaging 219 per year. Maintenance of a free zone and reliable export access to AHS-sensitive destinations in the face of sporadic outbreaks in surrounding areas is problematic, so that an alternative approach is required if horse exports from South Africa to AHS sensitive destinations are to be resumed on a regular and ongoing basis.

This paper describes the results of a quantitative risk assessment undertaken to estimate the probability of exporting an AHS-infected horse through a vector-protected quarantine facility in an infected country or zone, in accordance with OIE recommendations and allowing for additional biosecurity measures to provide further risk reduction.

## Materials and Methods

### Model overview

A stochastic simulation model was developed to estimate the probability of an undetected AHS-infected horse being exported from South Africa, under a variety of scenarios. The model was developed in the R statistical environment, version 3.1.1, [11]. All simulations were run for 10 000 iterations to produce probability distributions for all outputs. Outputs are presented as probability distributions of an individual undetected infected horse being exported, the expected number of horses per exported infected horse and the annual probability of an undetected infected horse being exported, assuming an annual export throughput of 300 horses.

The model simulated four main scenarios for a single exported horse from a vector-protected facility in either a low-risk area or from an endemically infected area elsewhere in South Africa, with multiple tests by real-time reverse transcription polymerase chain reaction assay (PCR) prior to and during pre-export quarantine and/or during post-arrival quarantine in the importing country, as summarised in Table 1.

**Table 1:** Summary of four main scenarios for an AHS quantitative risk model.

Scenario	1	2	3	4
**Scenario name**	LR.NoPAQ	LR.PAQ	EN.NoPAQ	EN.PAQ
**Risk area**	Low risk	Low risk	Endemic	Endemic
**PCR prior to pre-export quarantine**	Yes	Yes	Yes	Yes
**PCR during pre-export quarantine**	Yes	Yes	Yes	Yes
**Post-arrival quarantine and PCR**	No	Yes	No	Yes

LR, Low-risk area; EN, Endemic area; NoPAQ, No post-arrival quarantine or PCR; PAQ, Post arrival quarantine and PCR at destination; PCR, real-time reverse transcription polymerase chain reaction assay.

### Low risk area

For future exports and for the purposes of this study, the AHS low-risk area is defined as the free and surveillance zones, as defined in South African legislation, and is surrounded by the existing protection zone. For scenarios 1 and 2, additional risk mitigation measures in the low-risk area are assumed, based on existing control measures in this area, including: horse identification and census; compulsory vaccination in the protection zone and voluntary vaccination, subject to State veterinary approval, in the low-risk area; a mix of passive and active surveillance as defined in a surveillance plan, including monthly monitoring of sentinel horses using ELISA and PCR sufficient to provide 95% confidence of detecting a 2% prevalence of infected horses; additional unvaccinated sentinels in local horse populations in proximity to the quarantine facility; containment and elimination of outbreaks according to an existing response plan; and controls on movements of horses into the low-risk area and between zones within the low-risk area. As an additional precaution, vaccination in the low-risk area is being limited to the time of year with lowest vector activity, from June to October, to reduce the potential for possible vector transmission of vaccine-derived viruses.

### Vector-protected quarantine

All scenarios assume a fully enclosed, vector-protected facility, including filtered air intake, positive pressure ventilation, double door entry and exit and all horses, bedding, equipment and fodder would enter the facility at commencement of quarantine and remain there throughout, operating in accordance with a Veterinary Procedural Notice issued by South Africa’s Department of Agriculture, Forestry and Fisheries [12]. For the low-risk area, the facility is assumed to be located in an area of demonstrated low vector activity, as is currently the case for Kenilworth Quarantine Station.

Only staff and authorised visitors would be allowed access to the facility. Loading of horses into vector-protected jet stalls (mesh, insecticide and repellent) and loaded jet stalls into positive-pressure ventilated trailers would occur within the facility, before travel to the airport for loading. Loading would take place at a time determined by aircraft schedules, but would be co-ordinated to minimise potential exposure time during transhipment and undertaken during daylight hours where possible. Once loaded, the aircraft hold would be sealed and sprayed with insecticide prior to take-off.

Vector surveillance would be undertaken within the vector-protected facility, using standard ultra-violet light traps, running continuously and checked at least weekly. Any *Culicoides* spp. detections in the traps would trigger a restart of the quarantine period and associated testing.

For scenarios assuming PCR testing in post-arrival quarantine, a similar standard of facility is assumed.

### Additional risk management measures

For all scenarios, pre-export quarantine is assumed to be a minimum of 16 days in a vector-protected quarantine facility, with PCR tests five days prior to entering quarantine and two days prior to export, consistent with OIE recommendations for exports from an infected country or zone [6].

For scenarios from the low-risk area, a 40-day residency period in the low-risk area, including the quarantine period is assumed, whereas for scenarios from the endemic area, there are no additional risk management measures prior to export and no prior residency requirement. Scenarios that assume post-arrival quarantine include an additional PCR test, at least 14 days after arrival in the destination country, while in vector-protected post-arrival quarantine for at least 16 days.

A 40-day residency period was chosen for the low-risk area because this is consistent with the OIE’s infective period for AHS [6], so that any horse infected prior to entering the residency period would be expected to be either detected as infected, or no longer infectious, by the end of the residency period. This period was determined by the OIE Scientific Commission after considering all available data and is considered appropriate to ensure that a previously infected animal is no longer infectious. A similar risk period of 40 days prior to export was also used for scenarios from the endemic area for the same reasons as outlined above.

Sixteen-day pre-export and post-arrival quarantine periods were chosen because the OIE guidelines for exports from AHS-infected countries or zones using the agent identification method prescribe at least 14 days quarantine with an agent identification tests carried out no less than 14 days after entering quarantine and the expected turn-around time for receiving the PCR results was estimated to be 48 hours.

For all scenarios, a minimum 60-day residency period in South Africa prior to export is assumed and exported horses are not vaccinated within 40 days prior to export, to minimise the likelihood of vaccine-induced infections.

### Pathways for undetected infection in exported horses

**Fig 1:**
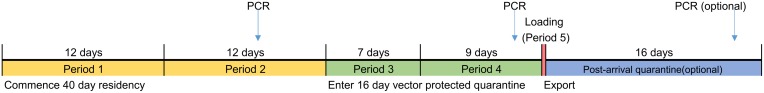
Timeline of risk periods for AHS infection in exported horses.

**Fig 2:**
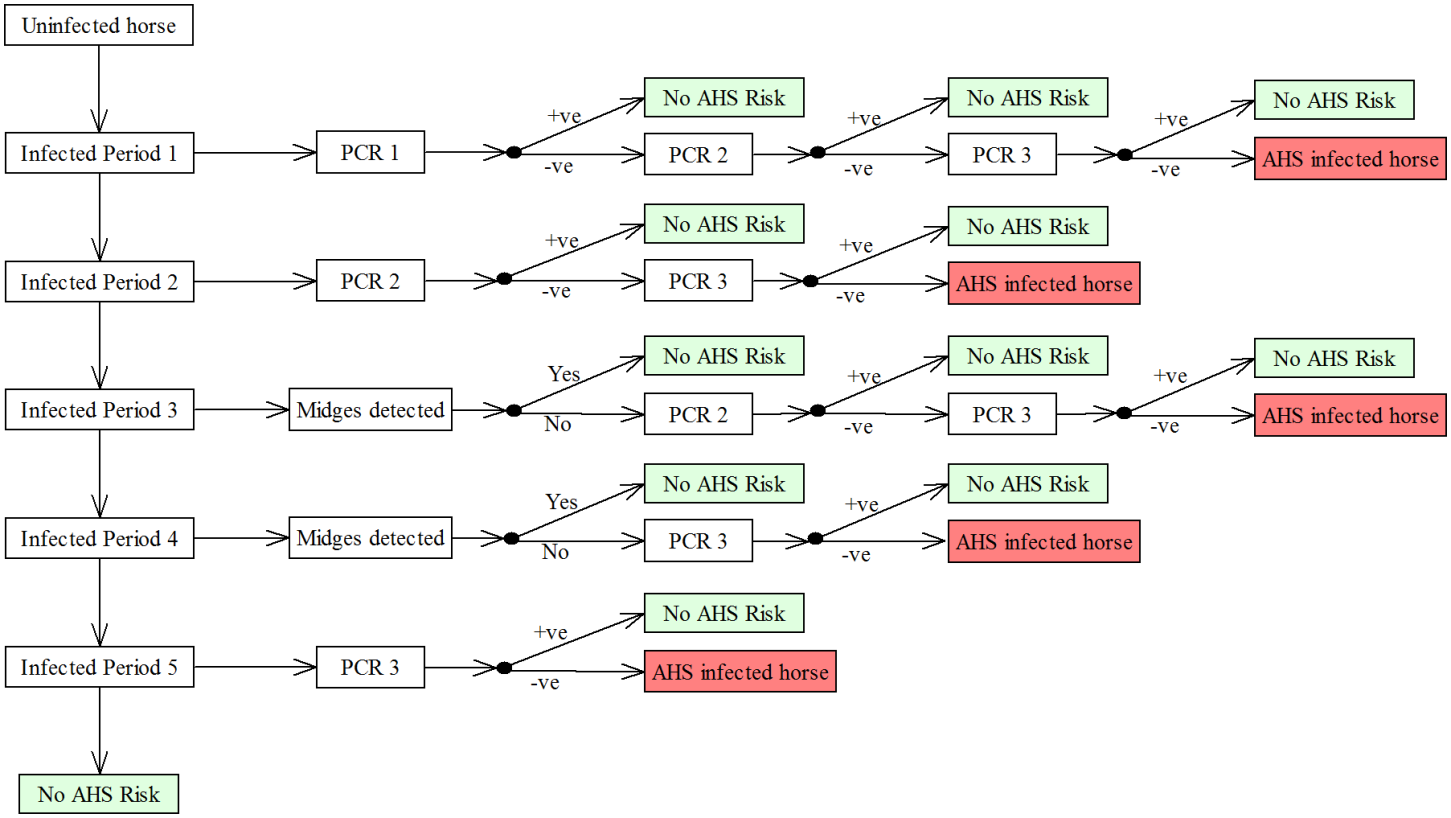
Scenario tree depicting pathways by which an undetected AHSV-infected horse could be exported from South Africa.

Five pathways were identified by which an undetected infected horse could be exported from South Africa. These pathways are summarised in Table 2 and graphically in Figs 1 and 2. Briefly, a horse could be infected in any one of five time periods identified in Fig 1 and then proceed to export undetected by the various PCR testing regimens applied. For horses infected during pre-export quarantine the relevant pathways assume a breakdown of vector protection, that the breakdown was not detected by vector surveillance within the facility and that infection was not detected by testing either at the end of quarantine or in post-arrival quarantine (where applicable). The time periods chosen reflect an assumption that PCR may not detect an incubating infected animal until seven or more days post-infection.

**Table 2:** Potential pathways for infection of horses for export of AHSV from South Africa.

Pathway	Description
**Pathway 1**	Infected in first 12 days of residency period, subject to two effective PCRs prior to export, prior to entering PEQ and immediately prior to export. Excluded for scenarios including PAQ, as horses infected via Pathway 1 no longer infectious.
**Pathway 2**	Infected in last 12 days before entry to PEQ, subject to one effective PCR immediately prior to export (PCR prior to entering PEQ ineffective due to timing). May be detected by PCR during PAQ, where applicable.
**Pathway 3**	Infected in first 7 days of quarantine, subject to one effective PCR immediately prior to export, not detected by vector surveillance. May be detected by PCR during PAQ, where applicable.
**Pathway 4**	Infected in last 9 days of quarantine, one PCR prior to export ineffective due to timing, not detected by vector surveillance. May be detected by PCR during PAQ, where applicable.
**Pathway 5**	Infected during trans-shipment at airport, no PCR or vector surveillance prior to export. May be detected by PCR during PAQ, where applicable.

PCR, real-time reverse transcription polymerase chain reaction assay; PEQ, pre-export quarantine; PAQ, Post-arrival quarantine.

For scenarios including PCR in post-arrival quarantine, an additional PCR was applied to all pathways, including Pathway 5. Also, for scenarios where post-arrival quarantine was included, Pathway 1 was excluded from the model, because the 40-day infective period had expired for these horses by the time they are released from post-arrival quarantine.

Probabilities associated with each pathway were combined multiplicatively, as described in the Model calculations section, to calculate a pathway probability for each pathway. Pathway probabilities were then summed across pathways to provide an overall probability of a single exported horse being infected and not detected and aggregated to produce an annual probability estimate, as described under Model calculations.

### Model inputs

Model inputs are summarised in Table 3 and described in more detail in the following sections.

**Table 3:** Input distribution and other parameters for an AHS quantitative risk model.

Input name	Description	Distribution	Source
*CFR*	Case-fatality rate	Beta(12, 64)	From 2014 outbreak (unpublished data)
*UnderReporting*	Multiplier for under-reporting of cases in endemic area	Pert(2, 3, 5)	Informed opinion of State epidemiologists with responsibility for collating notification data
*OutbreakFrequency*	Daily probability of an outbreak occurring	Gamma(outbreak duration, total days at risk for period)	Outbreak data for low-risk area, case notification for endemic area
*OutbreakIncidence*	Incidence rate for each outbreak (per horse-day at risk)	Gamma(estimated cases, horse-days at risk)	Outbreak data for low-risk area, case notification for endemic area
*Breakdown*	Daily probability of breakdown of vector protection	Gamma(24, 2191)	Unpublished data for existing facility, adjusted for imperfect detection of breakdowns
*Detection*	Probability a vector protection breakdown will be detected	Pert(0.2, 0.33, 0.5)	Informed opinion of experienced entomologists working with AHS
*LoadingBreakdown*	Probability of a vector protection breakdown during loading in the low-risk area	Gamma(1, 50)	Informed opinion of experienced entomologists working with AHS
*SePCR*	Sensitivity of RT-qPCR	Beta(9.65, 1.19) for lower sensitivity scenarios and Beta(185, 1.74) for higher sensitivity scenarios	Guthrie et al (2013)
*n*1, *n*2, *n*3, *n*4, *n*5	Time at risk for each of five risk periods	12 days, 12 days,7 days, 9 days, 2/24 hours = 0.083 days	See Fig 1
*PAR*	Population at risk	14 000 for low risk area; 268 000 for endemic area	Unpublished data from South African Department of Agriculture, Forestry and Fisheries

#### AHS incidence

A key input variable for the model is the probability of infection for a single horse either prior to entering pre-export quarantine or during the quarantine period. This was estimated as the *DailyRisk* (mean cases per horse-day at risk) of AHS based on existing data, adjusted for the efficacy of vector protection during the quarantine period.

For the low-risk area, there have been four well characterised outbreaks (1999, 2004, 2011 and 2014) in the surveillance zone since 1997, when the free zone was established (see Table 4). The 2013 outbreak was poorly characterised with only a small number of affected horses that were PCR positive and culture negative, with no accompanying signs indicative of AHS, and was excluded from these calculations. No other cases of AHS have been detected in the low-risk area despite extensive awareness among the horse industry and active surveillance to detect infection if it were present. For the 2011 and 2014 outbreaks a large number of horses in the outbreak area were tested by PCR and 84 and 74 cases were identified respectively, including a number of subclinical cases, which all tested positive on PCR and some on viral isolation, but showed no clinical signs of disease. For the 1999 and 2004 outbreaks, only deaths were recorded (32 and 16 respectively). However, data from the 2014 outbreak showed a 14.9% (11/74) case-fatality rate, allowing estimation of total numbers of cases for the 1999 and 2004 outbreaks.

**Table 4:** Numbers of reported cases by outbreak and outbreak duration for the low-risk area and by AHS season (year) for the endemic area.

Area	Year	Cases	Duration (days)
**Low-risk**	1999	32	57
	2004	16	56
	2011	84^a^	66
	2014	74^a^	73
**Endemic**	2005	540	210
	2006	562	270
	2007	83	300
	2008	904	240
	2009	426	300
	2010	196	240
	2011	1048	300
	2012	95	180
	2013	675	330
	2014	383	300

^a^ Total number of cases (clinical and sub-clinical recorded). Other outbreaks/years only deaths recorded.

*OutbreakIncidence* during an outbreak (the average number of cases per horse day at risk) for the low-risk area was modelled for the four recorded outbreaks as gamma distributions, with parameters of the observed or estimated number of cases during the outbreak and the number of horse-days at risk. For the 2011 and 2014 outbreaks actual numbers of cases recorded were used, while for the 1999 and 2004 outbreaks, the total number of *Cases* was estimated as:

Cases = Deaths / CFR (1)

where *Deaths* is the recorded number of deaths during the outbreak and *CFR* is the case-fatality rate, modelled as a Beta(12, 64) probability distribution, based on data from the 2014 outbreak. Horse-days at risk (*HDR*) was estimated as:

HDR = (PAR – Cases/2) × Duration (2)

Where *PAR* is the total population of the low-risk area (estimated at 14 000), *Cases* is the observed or estimated total number of cases for the outbreak and *Duration* is the duration of the outbreak in days. *Cases* is divided by 2 to subtract “time-at-risk” for half the cases, assuming that they occur on average mid-way through the follow-up period.

For the endemic area, *OutbreakIncidence* was estimated for each of the 10 years from 2005 to 2014, as a gamma distribution with parameters of the estimated number of cases for the year and the number of horse-days at risk (Table 4). The estimated number of cases each year was estimated based on reported number of notifications (deaths), adjusted for the estimated level of under-reporting and for assumed case-fatality rate, as:

Cases = Notifications × UnderReporting / CFR (3)

*UnderReporting* was modelled as a Pert(2, 3, 5), based on the informed opinion of State epidemiologists responsible for collating notification data, based on their consensus opinion of expected minimum, most likely, and maximum levels of under-reporting in the notifications data. *CFR* was modelled as a Beta(12, 64) distribution, as for the low-risk area. Horse days at risk was estimated using Eq 1, with *PAR* as the total population estimate for South Africa (282 000, unpublished data) less the estimated population of the low-risk area (14 000) and *Duration* is the number of months when cases were notified, multiplied by 30 days per month.

*OutbreakFrequency* (daily probability of an outbreak occurring) was modelled separately for each area as gamma a distribution. For the low-risk area, parameters were the sum of the durations of the four outbreaks and the total number of days at risk since the commencement of the free zone (18 years x 365 days) and for the endemic area parameters were the sum of the durations of outbreaks over the 10 years and the total number of days at risk (10 years x 365 days).

To account for variability among outbreaks, mean daily risk (*DailyRisk*) for each area was then modelled for each iteration of the model as the product of *OutbreakFrequency* and *OutbreakIncidence* for a randomly selected outbreak for the area.

#### Vector protection breakdown

The daily probability of breakdown of vector protection (*Breakdown*), so that potentially infected midges could enter the facility, was estimated from data for the Kenilworth facility between 2006 and 2011. Briefly, a total of eight midge detection events (1–3 midges each) occurred over 289 trapping events representing 2 191 trap-days. Assuming an average of 33% trap efficacy (informed opinion, see below, under *Vector breakdown detection*) for traps inside the facility this represents a 1.1% daily breakdown probability. The daily likelihood of breakdown of vector protection was modelled as Gamma(24, 2191) distribution, based on these data, but subjected to specific sensitivity analysis to evaluate the potential effect of improved vector protection in an upgraded facility.

#### Vector breakdown detection

The probability of detecting a breakdown of vector protection by light traps within the facility (*Detection*) was estimated as a pert distribution with minimum 0.2, mode of 0.33 and maximum 0.5. These estimates were based on the informed opinion of an entomologist with many years of experience working with AHS and *Culicoides* spp (G. Venter), who was asked to estimate the minimum most likely and maximum likelihoods of detecting a breakdown in vector protection by light traps in a closed facility in close proximity to horses, such as described here. The estimate reflects the view that light traps are not necessarily highly effective, depending on light and other environmental conditions at the time, as well as midge abundance, and that there is considerable uncertainty about their effectiveness, particularly when there are few midges present, as is expected to be the case if a breakdown of vector protection were to occur.

#### Breakdown of vector protection during loading

During loading, horses would be protected by application of an appropriate insect repellent (diethyltoluamide—DEET) prior to loading and covering of the jet stalls with insecticide-treated high-density polyethylene mesh before leaving the vector-protected quarantine facility. The hold of the plane would be sprayed with insecticide prior to loading and again after closure of the hold and before departure [12]. Page, et al. (2015) reported that use of alphacypermethrin-treated high-density polyethylene mesh significantly reduced numbers of midges recovered by aspiration from horses, but did not prevent midge attack [13]. This work was undertaken under conditions of high midge abundance and was run over 12 consecutive nights. The results should therefore be considered a worst-case scenario, particularly considering the duration of potential exposure and that concurrent use of repellent was not evaluated in that study. The results indicate that insecticide-treated mesh alone will reduce the frequency of midge attack, and hence the likelihood of exposure to AHS virus, and this likelihood would be further reduced in areas of low vector activity and with application of repellent to horses before loading. Based on these findings, the relatively short period of exposure (assumed to be <2 hours) and informed opinion of the entomologist identified above, the probability of breakdown of vector protection during loading (*LoadingBreakdown*) was modelled as a Gamma(1, 50) distribution, with a mean value of 2%, and was also subjected to further sensitivity analysis. The subject matter expert was asked to estimate the likely frequency (number of trials per occurrence) of breakdowns during loading under the conditions described.

#### Sensitivity of PCR

The sensitivity of PCR (*SePCR*) was estimated from published research by Guthrie, et al. [14]. Briefly, their work presented estimates for sensitivity of an RT-qPCR for AHSV, in horses exhibiting one or more clinical signs of AHS. The estimate chosen for this analysis was estimated from a model that allowed for lack of independence with the comparison test of virus isolation and included informative priors for the specificity of virus isolation and prevalence in an AHS-free population (Model 1). This estimate had a median value of 0.978 and 95% interval of 0.708 to 0.9996. This estimate has the benefits of being conservative, with quite a long tail, allowing for the possibility of lower sensitivity in subclinical cases or where tests are repeated at relatively short intervals. Parameters for a Beta probability distribution were estimated from the above estimates, with an assumed mode of 0.978 and fifth percentile of 0.708 [15].

### Model calculations

The probabilities of an undetected infected horse being exported via Pathways 1 to 5 (*P*1 to *P*5 respectively) for scenarios assuming no post arrival quarantine or PCR were calculated as:

$$P1=[1-\;{\left(1-DailyRisk\right)}^{n1}\;\times \;{\left(1-SePCR\right)}^{2}$$

$$P2=\left(1-P1\right)\;\times \left[1-\;{\left(1-DailyRisk\right)}^{n2}\right]\times \left(1-SePCR\right)$$

$$P3=\left(1-P1\right)\times \left(1-P2\right)\times \left[1-\;{\left(1-DailyRisk\;\times Breakdown\right)}^{n3}\right]\times \left(1-Detection\right)\times \left(1-SePCR\right)$$

$$P4=\left(1-P1\right)\times \left(1-P2\right)\times \left(1-P3\right)\times \left[1-\;{\left(1-DailyRisk\;\times Breakdown\right)}^{n4}\right]\times \left(1-Detection\right)$$

P5=(1−P1)×(1−P2)×(1−P3)×(1−P4)×P5 ×DailyRisk ×LoadingBreakdown

Where *n*1 to *n*5 are the duration in days for each of the risk periods 1 to 5 respectively (see Fig 1). For scenarios including post-arrival quarantine, *P*1 = 0 and *P*2 to *P*5 were multiplied by an additional (1 –*SePCR*) to allow for the additional PCR during post-arrival quarantine.

The overall probability of an exported horse being infected and undetected is then calculated as the sum of the probabilities for the five pathways.

PHorse = P1 + P2 + P3 + P4 + P5

This overall probability was then converted to the expected numbers of horses exported per infected horse (= 1/*PHorse*) and an annual probability of one or more undetected infected horses being exported assuming a total of 300 horses exported per year:

$$PAnnual=1-\;{\left(1-PHorse\right)}^{300}$$

The proportional contribution of each pathway to the overall probability was also calculated, to determine which pathways contributed most to the overall risk for each scenario.

### Sensitivity analysis

A sensitivity analysis was undertaken to identify the most influential variables on *PHorse* for each scenario, using visual inspection of tornado plots.

Four additional scenarios were also run to evaluate the effect of assuming a higher and less uncertain distribution for the sensitivity of PCR, based on Model 3 from Guthrie, et al. (2013). These scenarios were the same as the four base scenarios, except that a Beta(185, 1.74) probability distribution was used for *SePCR*, with a mode of 0.996 and fifth percentile of 0.977.

Finally, additional scenarios were run to evaluate the importance of vector protection in the model, using values for *Breakdown* varying from 1/5 000 to 1/5, modelled as gamma distributions, with all other variables as for Scenarios 1 and 3. Similarly, scenarios were run using values for *LoadingBreakdown* ranging from 1/500 to 1/5, modelled as gamma distributions and also compared to base Scenarios 1 and 3.

## Results

### AHS incidence

The median *OutbreakFrequency* for the low-risk area was 3.8% of days (95% predictive interval (PI): 3.4%–4.3%) and for the endemic area 73.1% (95% PI: 70.4%–75.9%). Median incidence during outbreaks (*OutbreakIncidence*) was 369 cases per 10 000 horse-years at risk (95% PI: 227–1380) for the low-risk area and 453 cases per 10 000 horse-years (95% PI: 62–1562) for the endemic area. Median overall *DailyRisk* was 14.3 cases per 10 000 horse-years at risk (95% PI: 8.5–53.4) for the low-risk area compared to 331 cases per 10 000 horse-years at risk (95% PI: 45–1134) for the endemic area.

### Probability of exporting an AHS infected horse

The median probabilities and 95% predictive intervals of undetected infection in a single exported horse and the annual probability of one or more undetected infected horses being exported, assuming an annual throughput of 300 horses, are summarised for the main scenarios in Table 5. Briefly, the median probability of an exported horse being infected and not detected prior to export was 5.4 x 10^−6^ (equivalent to one undetected infected horse in every 187 000 horses exported) for scenario LR.NoPAQ, from the low-risk area. Inclusion of post-arrival quarantine and PCR at the destination reduced the median probability of an undetected introduction by approximately 12-fold, to 4.6 x 10^−7^ (equivalent to one undetected infected horse in every 2.2 million horses exported) for scenario LR.PAQ. The median probability of exporting an undetected infected horse from the endemic area was 15 to 17 times higher than for the low-risk area for comparable scenarios EN.NoPAQ and EN.PAQ.

**Table 5:** Median and 95% predictive limits for probability of undetected AHS-infection for a single exported horse and annual probability of one or more undetected infected horses being exported.

	Probability of undetected infection for a single exported horse			Annual probability of one or more undetected infected horses being exported^a^		
Scenario	50%	2.50%	97.50%	50%	2.50%	97.50%
**LR.NoPAQ**	0.000005346	0.000000510	0.000041248	0.001602440	0.000153004	0.012298243
**LR.PAQ**	0.000000461	0.000000003	0.000008860	0.000138204	0.000000976	0.002654456
**EN.NoPAQ**	0.000092171	0.000005771	0.000809982	0.027273604	0.001729778	0.215801453
**EN.PAQ**	0.000007150	0.000000043	0.000192613	0.002142666	0.000012756	0.056151264

LR, Low-risk area; EN, Endemic area; NoPAQ, No post-arrival quarantine or PCR; PAQ, Post arrival quarantine and PCR at destination; PCR, real-time reverse transcription polymerase chain reaction assay.

^a^ Assumes 300 horses exported per year.

### Importance of different pathways

**Fig 3:**
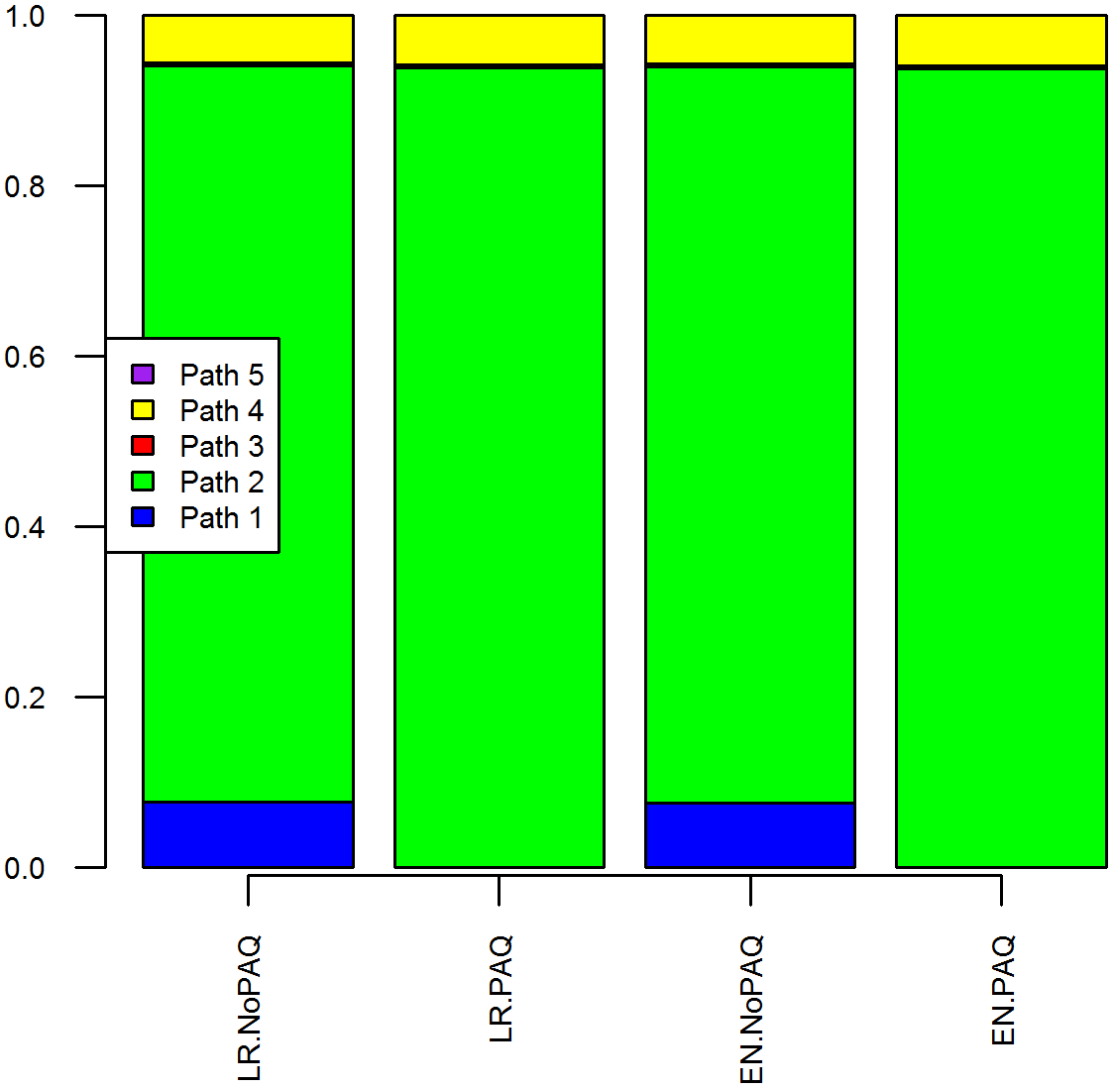
Proportional contribution of different pathways to median overall probability of exporting an undetected infected horse. LR, Low-risk area; EN, Endemic area; NoPAQ, No post-arrival quarantine or PCR; PAQ, Post arrival quarantine and PCR at destination; PCR, real-time reverse transcription polymerase chain reaction assay.

For all scenarios, Pathway 2 (infected in the last 12 days prior to entering pre-export quarantine) was responsible for the majority contribution to the overall probability of exporting an infected horse (Fig 3). Pathways 3 (infected during the first seven days of quarantine) and 5 (infected during loading) provided negligible contribution to overall probability of undetected infection. Pathway 1 made no contribution to risk for scenarios including post-arrival quarantine, because the post-arrival quarantine period nullified any risk associated with infection during this period.

### Scenarios assuming higher PCR sensitivity

**Fig 4:**
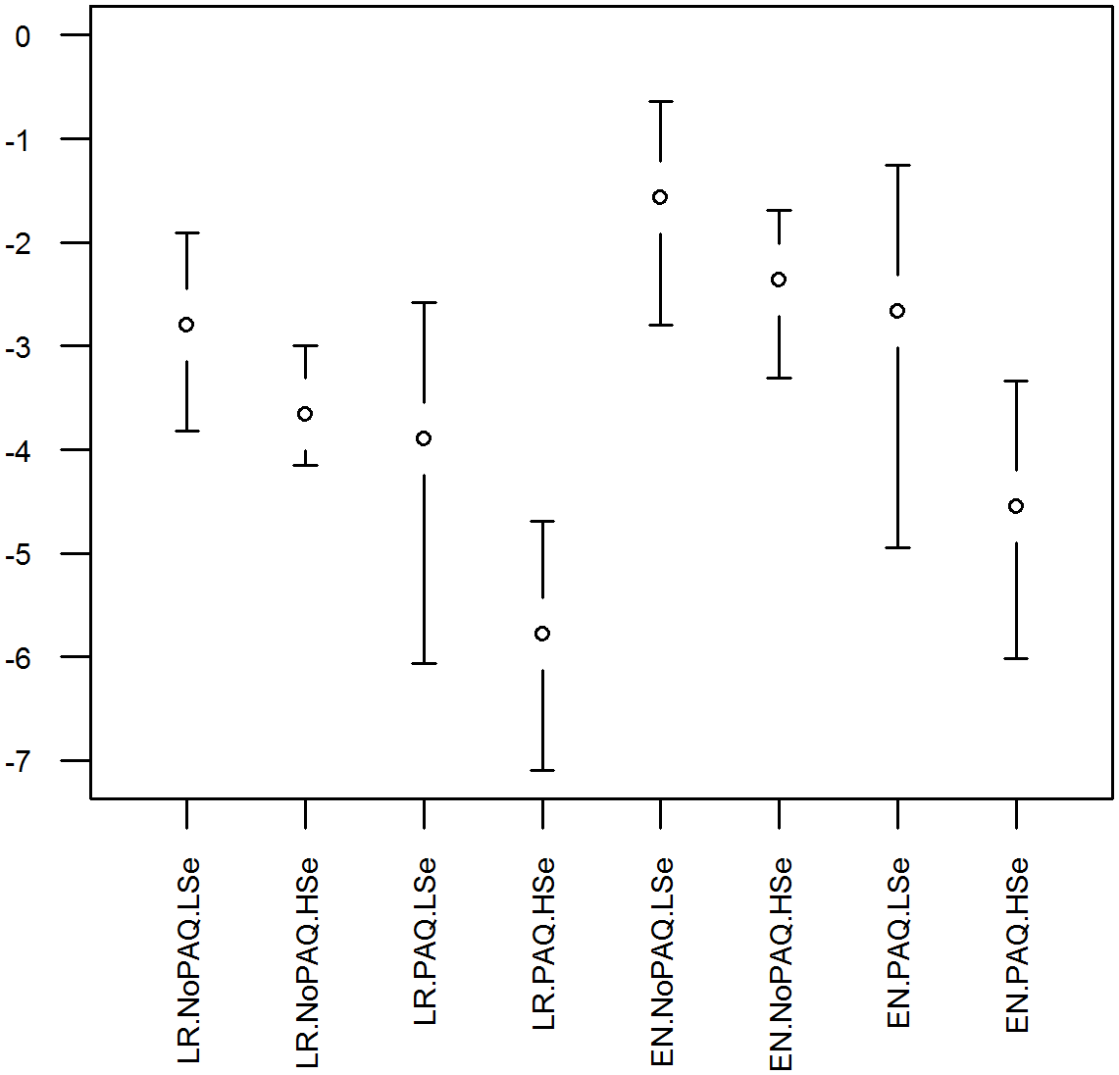
Median and 95% predictive intervals for the log (base 10) of annual probability of exporting one or more undetected AHS-infected horses. Assumes 300 horses exported per year. LR, Low-risk area; EN, Endemic area; NoPAQ, No post-arrival quarantine or PCR; PAQ, Post arrival quarantine and PCR at destination; PCR, real-time reverse transcription polymerase chain reaction assay; LSe, lower PCR sensitivity estimate used; HSe, higher PCR sensitivity estimate used.

Scenarios assuming a higher-sensitivity of PCR reduced the probability of exporting an infected undetected horse by approximately 7-fold for scenarios assuming no post-arrival quarantine and PCR and 77-fold for scenarios including post-arrival quarantine and PCR, compared to scenarios assuming the lower and more uncertain distribution (Fig 4). The reduced uncertainty about PCR sensitivity in these scenarios also reduced uncertainty in model outputs, resulting in a narrower predictive interval compared to other scenarios.

### Sensitivity analysis

**Fig 5:**
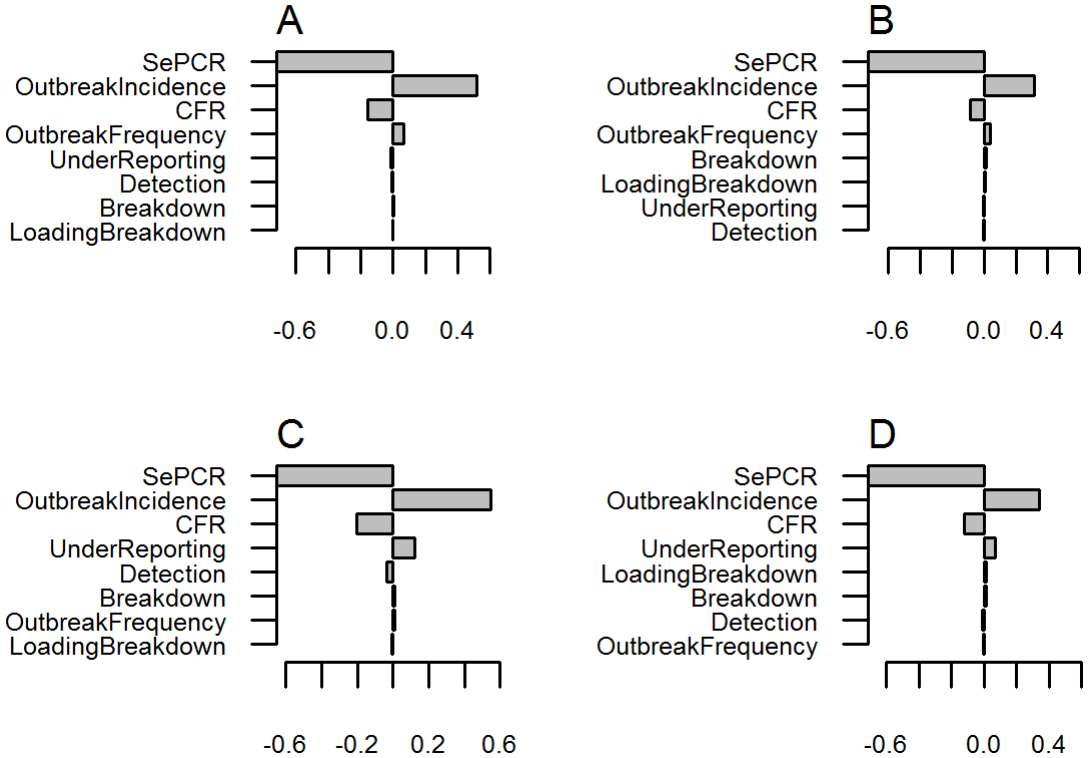
Tornado plots of correlation coefficients for input variables and probability of exporting an undetected infected horse. A, low-risk area with no post-arrival quarantine; B, low-risk area with post-arrival quarantine and PCR; C, endemic area with no post-arrival quarantine; D, endemic area with post-arrival quarantine and PCR.

For all scenarios, *SePCR* sensitivity was the most influential input in the model, followed by *OutbreakIncidence* and case-fatality rate (*CFR*), as shown in Fig 5. *OutbreakFrequency* had a minor impact for scenarios from the low-risk area but not from the endemic area. Conversely, *UnderReporting* was a small contributor for scenarios from the endemic area, but not the low-risk area, where this input was not used. Other inputs relating to vector protection breakdown had negligible influence on model outputs.

#### Breakdown of vector protection

Varying the probability of breakdown of vector protection from 1/5000 (0.02%) to 1/10 (10%) per day had only minimal effect on the annual probability of exporting an undetected infected horse, compared to the base scenario, until the probability of breakdown was around 2% per day or greater, for scenarios from both the low-risk and endemic areas (see S2 Fig). Similarly, varying the probability of breakdown of vector protection at loading from 1/500 (0.2%) to 1/5 (20%) had no noticeable effect on the annual probability of exporting an undetected infected horse, even up to 20% probability of breakdown (see S3 Fig).

## Discussion

To our knowledge this is the first time a quantitative analysis of the probability of exporting undetected AHS-infected horses from an infected country has been undertaken. Two previous risk assessments for AHS were identified, relating to the risk of importing AHS into The Netherlands and France, respectively, with imported equids from a variety of sources of varying risk levels [16, 17]. The approach taken in those assessments was quite different to the analysis presented here, in that the analyses quantified risk associated with imports, based on existing data of numbers of equids imported from countries with varying risk levels and including wild equids as well as horses. The analysis also assumed screening with serology rather than PCR and a longer quarantine period. As a result, the results of that analysis are not directly comparable to the current analysis. Interestingly that assessment did include imports of small numbers of horses from identified “high risk” regions. However, their contribution to the overall risk estimate was small, because of larger numbers of other equid species from high risk regions, as well as a higher overall contribution from low-risk regions because of larger numbers of imports.

The OIE Code recommends the use of vector-protected quarantine and testing as an appropriate measure for risk management for export of live horses from infected countries [6]. However few countries have been prepared to accept exports from South Africa under these guidelines, other than via the free zone, presumably because of the perceived risk associated with the possible breakdown of vector protection and lack of back-up risk mitigation measures.

The results presented here clearly demonstrate that it is possible to manage the risk of AHS infection in horses exported from an infected country or zone. The model allows for the application of multiple layers of risk management, including external risk mitigation through use of prior residency in an area of demonstrated low-risk of AHS, vector-protected pre-export quarantine, PCR testing to detect infection and, optionally, post-arrival quarantine and further testing in the destination country. The proposed low-risk area has a long history of demonstrated low incidence of AHS, with only four confirmed outbreaks and an estimated total of about 480 infected horses over the 18 years since the original free zone was established.

The probability of exporting undetected infected horses could be further reduced, effectively to zero, by limiting exports to periods when there are no outbreaks. This would be achievable in the low-risk area and would still allow exports to proceed unimpeded in most years and for the majority of the year when an outbreak does occur. However, this might be more difficult for exports from the endemic area where, on average, outbreaks occur in nine months of any given year.

The occurrence of undetected outbreaks in the low-risk area would result in an increase in the probability of exporting an undetected infected horse. However, there is a high awareness of AHS in the low-risk area, along with movement controls and active and passive surveillance, so that an outbreak is highly unlikely to remain undiscovered for more than a short period of time, so that the outbreak frequency and incidence figures used for the low-risk area are considered appropriate.

Ongoing horse census, movement controls, surveillance and research provide substantial support for the maintenance of the low-risk area, with any residual risk further reduced by location of the vector-protected quarantine facility in an area of demonstrated low vector activity and use of PCR testing prior to and during pre-export quarantine. For some destinations, two PCR tests, prior to entry into pre-export quarantine and towards the end of the quarantine period, may be sufficient to adequately manage the risk. For more risk-averse destinations, an additional PCR test during post-arrival quarantine would further reduce the risk of introducing AHS by approximately 7-fold.

A residency period of 40 days in the low-risk area was considered a critical part of risk management for AHS. Forty days is the infective period for AHS as defined by the OIE, so that any recently infected horses from the endemic area entering the low-risk area would be expected to have cleared the infection and no longer be infectious by the time of export, regardless of any quarantine or testing that might be applied. Although it is possible that infection might persist longer than the 40-day period, there is no evidence to support this and the OIE determination was based on the best data available to the Scientific Commission at the time, so this assumption is considered appropriate.

A proposed requirement to only vaccinate horses during periods of low vector activity (June to October) was another important risk mitigation measure for the low-risk area. The occurrence of vaccine infections and potential for transmission of vaccine strains, reversion to virulence or reassortment of vaccine viruses to new, virulent strains cannot be ignored. For example, recent studies have shown that live attenuated vaccines have “repeatedly reassorted with field strains, contributing to their genotypic, and potentially phenotypic, variability” [18]. Similarly, reassortment of attenuated vaccine strains has also been suspected in the evolution of novel virulent strains of avian influenza virus in Taiwan [19]. By limiting vaccination to periods of low vector activity, the likelihood of these events occurring is minimised, as cases of AHS have never been recorded in the low-risk area during this period of the year.

The overall risk of exporting an undetected AHS-infected horse from the endemic area was approximately 15 to 17 times that for equivalent scenarios from the low-risk area, largely due to the increased frequency of AHS outbreaks and the corresponding lack of additional risk mitigation measures. However, this risk could again be further reduced by applying an additional PCR test during post-arrival quarantine.

One additional risk mitigation measure that was not included in the model was the daily inspection of animals and checking for clinical signs of disease. Clinical signs were not included in the model because an animal with ongoing clinical signs is likely to be detected as positive on PCR test either as part of the investigation or prior to export. Conversely, an animal that shows transient clinical signs and is negative on PCR will be allowed for export provided it is healthy at the time of loading. Therefore the only horses to be excluded from export solely on the basis of clinical signs would be those that are PCR negative and that are still showing signs at the time of export. The likelihood of this was considered to be sufficiently low to not warrant inclusion in the model.

The possibility of escape of AHSV from post-arrival quarantine was also not included in the model, for relevant scenarios. This was excluded from the model because it would require a breakdown of vector protection sufficient for one or more midges to enter the facility, feed on an infected horse, escape from the facility and subsequently feed on a susceptible horse. Post-arrival quarantine is the responsibility of the importing country and therefore beyond the capability of South African Authorities to control. Further, assuming that the facility is of a similar standard to the existing pre-export facility, the likelihood of midges entering and subsequently escaping was considered sufficiently low to be ignored for the purpose of this model.

An important driver of the model was outbreak frequency for AHS in the population of origin of horses for export and in the vicinity of the export facility. For the low-risk area, outbreak frequency was very low, at about 3% of days, based on estimates from comprehensive data on previous outbreaks, compared to about 73% of days for the endemic area, based on National notifications data. This difference in outbreak frequency was the main difference between the low-risk and endemic areas and highlights the advantage of maintaining a low-risk area as part of a multi-faceted risk management strategy for prevention of AHS.

Incidence during outbreaks for the low-risk area was also based on data for four well-documented outbreaks, whereas estimated incidence for the endemic area was based on case notifications, adjusted for estimated level of under-reporting and a case-fatality rate estimated from one outbreak in the low-risk area. This case fatality rate was low compared to quoted estimates for virulent AHS infections [1–3] and so may over-estimate the true incidence of AHS during outbreaks. If this were the case, the resulting probability of exporting an infected horse would be further reduced. Alternatively, strains of AHS occurring periodically in the low-risk area may be less virulent than elsewhere.

A second key driver was the assumed sensitivity of the PCR test. This was based on previously published data on an assay that is available for routine use in South Africa. The median estimate of sensitivity for this test was quite high at 97.8%. However, the 95% interval around the estimate was very wide, with a lower limit of 70.8%. This uncertainty contributed to the very wide probability limits around the risk estimates for the current model, as shown in the sensitivity analysis. While it can be argued that the estimate used is appropriate and allows for a lower sensitivity in subclinical cases, recent data on subclinically infected horses has shown PCR Ct values that are well above the limits of detection for the test [5]. It is therefore feasible that the estimates used in this study underestimate the true sensitivity in subclinical cases. Additional analyses showed that the probability of exporting an undetected infected horse could be substantially reduced if a higher and less uncertain sensitivity of PCR could be assumed. An additional PCR test, for example at day 7 of pre-export quarantine, could also be used to further reduce the overall risk, but was not included in the model because of the potential for correlation of test results, as discussed below.

An alternative PCR for the detection of AHS was published in 2008 and could provide an alternative to the PCR used for this analysis [20]. Diagnostic sensitivity for this test in a recent evaluation was 97% based on 128 samples testing positive from a panel of 132 known positive samples (Aguero-Garcia and Castillo-Olivares, personal communication). This would result in probability estimates for an undetected infected horse that are intermediate between the results for the two sensitivity estimates from Guthrie et al. 2013.

The proposed risk management measures included in the model include up to three PCR tests at intervals of 19 and 16 days. This leads to a potential concern that test results could be correlated, so that the sensitivity of subsequent PCR tests might be over-estimated. We do not believe that this is a genuine concern, for a number of reasons. Briefly, correlation of test results is only an issue in this analysis if an infected animal gives repeated negative results, due either to a low-level viraemia in incubating or convalescent phase of disease or to inhibition of the PCR reaction. PCR inhibition is not an issue for the assay under consideration, due to the inclusion of a positive control in all assays undertaken. If inhibition occurred, this would be detected by failure of the positive control and testing would be repeated. In relation to incubating animals, the incubation period for AHS is generally less than seven days, but occasionally longer. The timing of testing is such that there are at least two incubation periods between tests, so that an incubating animal might be negative at one test but is highly unlikely to be negative again at subsequent tests. On the other hand, the PCR is able to detect AHSV RNA in vaccinated or convalescent animals that are thought to no longer be infectious [21], so that correlation of negative results in convalescent animals is again unlikely to be a concern. Finally, the distribution used for PCR sensitivity has a very wide 95% interval, indicating considerable uncertainty about the true value. This distribution was based on a Bayesian analysis using appropriately informed priors and was therefore considered to be the most appropriate distribution to use. However, the long tail of the distribution is not consistent with field experience of the assay, which is that it is a highly sensitive test in natural infections. Accordingly, we believe that the degree of uncertainty expressed in this distribution provides additional safeguards against any concerns of potential correlation.

An important finding of this analysis was that the efficacy of vector protection during quarantine was generally not influential in the model. In fact, for the base scenarios, the probability of breakdown of vector protection had only minor effects at values less than 2% per day.

One previous study considered the effect of the probability of breakdown in vector protection on the probability of infection of animals with the related bluetongue virus [22]. The authors of that study found it was “not possible to directly estimate the effectiveness of this condition given the scarce available data”. They also did not attempt to specify or describe what constitutes “vector protection” and therefore used arbitrary estimates of the effectiveness of vector protection ranging from 50% to 100%. In contrast, the estimates used in our study were based on data from an existing facility, recognising that the proposed new facility is expected to have a higher level of protection.

The model presented here assumes ongoing vector surveillance within the vector-protected facility, to detect breakdowns if they occur. Estimating the probability of detection of midges is challenging as it depends on many factors, including the type of trap used, local environment, ambient light, local abundance of the midge and the proximity to the host. For this model we assumed that the detection of midge incursions inside a vector-protected facility is generally likely to be relatively poor, particularly considering that breakdowns in a highly biosecure facility are likely to result in only low numbers of midges gaining entry, reducing the likelihood of detecting midges in the traps. In fact, the sensitivity analysis undertaken showed that this was not an influential variable in the model.

The efficacy of vector protection at loading was also difficult to estimate. However, considering that protection during loading was limited to application of repellent and use of insecticide impregnated mesh, the likelihood of breakdown was considered to be higher than for the fully enclosed vector-protected facility. Loading during daylight hours and minimising the exposure period during loading are additional risk mitigation measures that would be beneficial, but are affected by aircraft schedules and logistic issues that are often out of the control of the exporter and hence weren’t considered further in this analysis, other than to assume proximity to a major airport and a two hour window for loading of pre-loaded jet stalls into the aircraft. Regardless of the difficulty in estimating an appropriate value for the probability of breakdown of vector protection during loading, sensitivity analysis of this variable also showed that the actual value is not critical and had no impact on model output for values up to 20%.

An important potential contributor to overall risk is the possibility of stop-over by the aircraft in another AHS-infected country to load or unload other freight. This is not specifically included in the model and it is assumed that this risk is minimised by maintaining vector-protection on the horses and jet stalls throughout the journey and by spraying the aircraft hold with insecticide following any stop-overs. For highly risk averse countries, any residual risk due to stop-overs could be addressed by requiring post-arrival quarantine and PCR testing at destination.

The estimates produced by this model have considerable uncertainty associated with them, reflecting the uncertainty in many of the input values, in particular the sensitivity of PCR and incidence during outbreaks. Reducing uncertainty about these estimates could substantially reduce the uncertainty in the outputs and improve the precision of the model. However, in the absence of more definitive estimates, the current values provide a useful model that can be used to objectively evaluate the probability of exporting undetected infected horses under a variety of scenarios.

## Conclusion

The results of this research clearly demonstrate that the risk of AHS infection in horses exported from an infected country can be minimised by appropriate risk management measures. Critical components of risk management for AHS are a vector-protected quarantine facility for pre-export quarantine, supported by pre-export testing using recommended tests for AHS. However, additional risk management may be required to provide backup in case of a breakdown in vector protection, in the form of either pre-border measures, based on a demonstrated low risk of infection prior to and during pre-export quarantine, or post-border by the use of vector-protected post-arrival quarantine and additional testing.

## Supporting Information

S1 Dataset Simulation results for base scenarios. (ZIP)

S2 Dataset Simulation results for comparison of high and low PCR sensitivity. (ZIP)

S3 Dataset Simulation results for sensitivity analysis on breakdown of vector protection during quarantine. (ZIP)

S4 Dataset Simulation results for sensitivity analysis on breakdown of vector protection during loading. (ZIP)

S1 Fig Map of AHS Controlled Area of South Africa. (PDF)

S2 Fig Results of sensitivity analysis on the probability of breakdown of vector protection during pre-export quarantine. A, Low-risk area, *Breakdown* = gamma(24, 2191); B, Low-risk area, *Breakdown* = gamma(1, 5000); C, Low-risk area, *Breakdown* = gamma(1, 500); D, Low-risk area, *Breakdown* = gamma(1, 50); E, Low-risk area, *Breakdown* = gamma(1, 10); F, Low-risk area, *Breakdown* = gamma(1, 5); G, Endemic area, *Breakdown* = gamma(24, 2191); H, Endemic area, *Breakdown* = gamma(1, 5000); I, Endemic area, *Breakdown* = gamma(1, 500); J, Endemic area, *Breakdown* = gamma(1, 50); K, Endemic area, *Breakdown* = gamma(1, 10); L, Endemic area, *Breakdown* = gamma(1, 5).
(TIFF)

S3 Fig Results of sensitivity analysis on the probability of breakdown of vector protection during loading. A, Low-risk area, *LoadingBreakdown* = gamma(1, 500); B, Low-risk area, *LoadingBreakdown* = gamma(1, 50); C, Low-risk area, *LoadingBreakdown* = gamma(1, 10); D, Low-risk area, *LoadingBreakdown* = gamma(1, 5); E, Endemic area, *LoadingBreakdown* = gamma(1, 500); F, Endemic area, *LoadingBreakdown* = gamma(1, 50); G, Endemic area, *LoadingBreakdown* = gamma(1, 10); H, Endemic area, *LoadingBreakdown* = gamma(1, 5).
(TIFF)
