# Supplementary material for: Quantitative Risk Assessment for African Horse Sickness in Live Horses Exported from South Africa
Source: PLoS One. 2016 Mar 17;11(3):e0151757. doi: 10.1371/journal.pone.0151757 (PMC4795756; doi:10.1371/journal.pone.0151757)
Supplement: S1 Fig — (PDF) [file pone.0151757.s005.pdf]

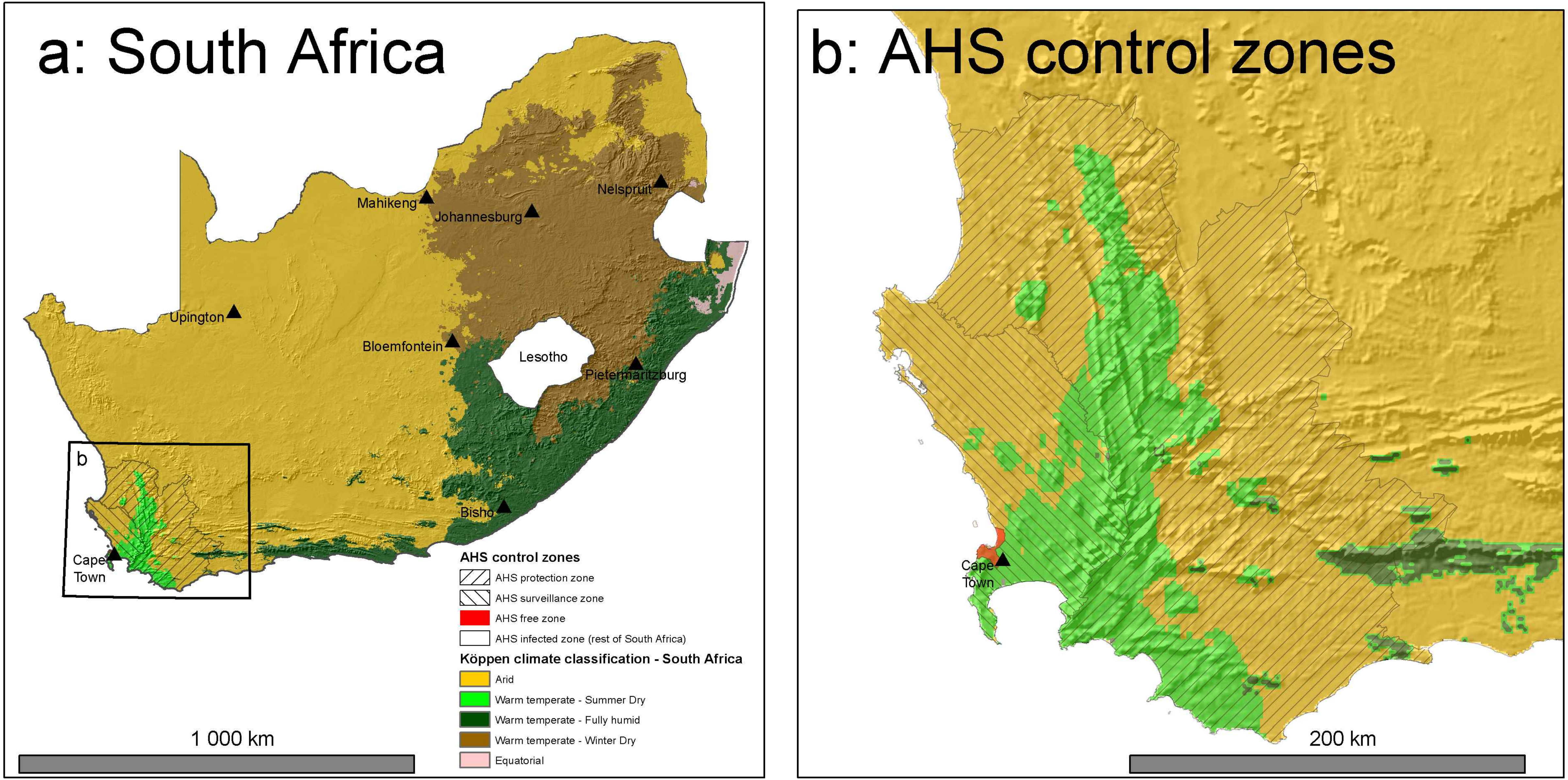

a: South Africa
Mahikeng
Johannesburg
Nelspruit
Upington
Bloemfontein
Lesotho
Pietermaritzburg
Bisho
Cape Town
b
AHS control zones
AHS protection zone
AHS surveillance zone
AHS free zone
AHS infected zone (rest of South Africa)
Köppen climate classification - South Africa
Arid
Warm temperate - Summer Dry
Warm temperate - Fully humid
Warm temperate - Winter Dry
Equatorial
1 000 km
b: AHS control zones
Cape Town
200 km
