# Supplementary figures and images for: Quantitative Risk Assessment for African Horse Sickness in Live Horses Exported from South Africa
Source: PLoS One. 2016 Mar 17;11(3):e0151757. doi: 10.1371/journal.pone.0151757 (PMC4795756; doi:10.1371/journal.pone.0151757)

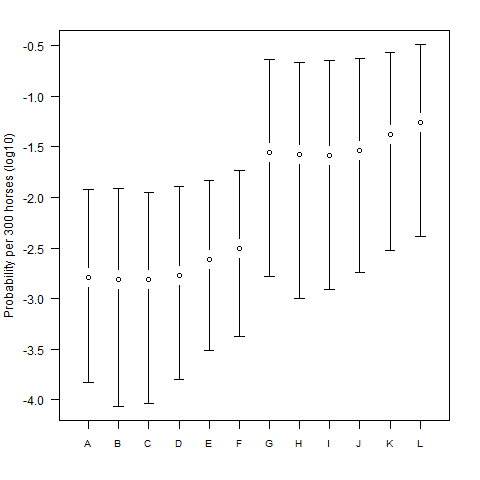

Supplement: S2 Fig — A, Low-risk area, Breakdown = gamma(24, 2191); B, Low-risk area, Breakdown = gamma(1, 5000); C, Low-risk area, Breakdown = gamma(1, 500); D, Low-risk area, Breakdown = gamma(1, 50); E, Low-risk area, Breakdown = gamma(1, 10); F, Low-risk area, Breakdown = gamma(1, 5); G, Endemic area, Breakdown = gamma(24, 2191); H, Endemic area, Breakdown = gamma(1, 5000); I, Endemic area, Breakdown = gamma(1, 500); J, Endemic area, Breakdown = gamma(1, 50); K, Endemic area, Breakdown = gamma(1, 10); L, Endemic area, Breakdown = gamma(1, 5). (TIFF) [file pone.0151757.s006.tiff]

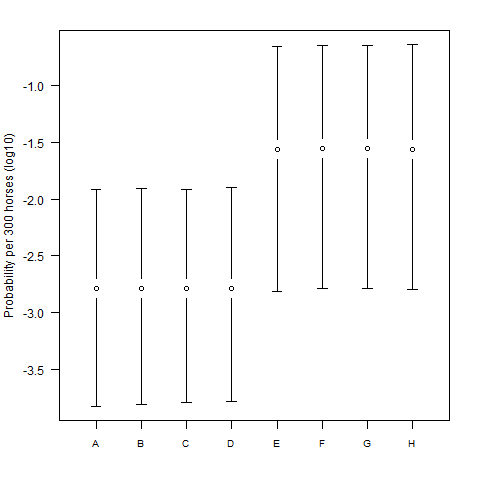

Supplement: S3 Fig — A, Low-risk area, LoadingBreakdown = gamma(1, 500); B, Low-risk area, LoadingBreakdown = gamma(1, 50); C, Low-risk area, LoadingBreakdown = gamma(1, 10); D, Low-risk area, LoadingBreakdown = gamma(1, 5); E, Endemic area, LoadingBreakdown = gamma(1, 500); F, Endemic area, LoadingBreakdown = gamma(1, 50); G, Endemic area, LoadingBreakdown = gamma(1, 10); H, Endemic area, LoadingBreakdown = gamma(1, 5). (TIFF) [file pone.0151757.s007.tiff]
